# Does the education system serve as a persuasion agent for recommending ADHD diagnosis and medication uptake? A qualitative case study to identify and characterize the persuasion strategies of Israeli teachers and school counselors

**DOI:** 10.1186/s12888-019-2120-9

**Published:** 2019-05-17

**Authors:** Anat Gesser-Edelsburg, Rasha Hamade Boukai

**Affiliations:** 1 School of Public Health, University of Haifa, 199 Aba Khoushy Ave. Mount Carmel, 3498838 Haifa, Israel; 2 The Health and Risk Communication Research Center, University of Haifa, 199 Aba Khoushy Ave. Mount Carmel, 3498838 Haifa, Israel

**Keywords:** ADHD diagnosis, Medication uptake, Persuasion strategies, Teachers and school counselors, Discourse with parents, Qualitative study

## Abstract

**Background:**

There has been a steady rise in the use of medication by Israeli school children to treat ADHD, partly due to what seems like school teachers’ and counselors’ tendency to express positive attitudes towards its use. Therfore it is important to examine the involvement of the school teachers and counselors in the parents’ decision-making about giving their children medication.

**Methods:**

This study used a qualitative constructivist research method of semi-structured interviews. It included individual interviews with 36 teachers and school counselors and 11 parents of students ages 9–14 from the Jewish and Arab populations.

**Results:**

Teachers and school counselors use different strategies to encourage parents to have their children diagnosed for ADHD and medicated. First they suggest diagnosis as a necessary step in the best interest of the child, distinguishing between diagnosis and medication to mitigate parents’ concerns. In the second stage, teachers normalize the use of medication, as well as framing it as a drug that provides not only a medical treatment but also emotional wellbeing.

**Conclusions:**

Teachers and counselors are involved in parents’ decision-making process about medicating their children to treat ADHD, which contradicts the education system’s guidelines. It is necessary to set clear and explicit limits and guidelines for education system employees so that they do not cross professional and ethical limits.

## Background

Attention Deficit Hyperactivity Disorder (ADHD) is the most common neurobehavioral disorder in children [1, 2]. There is wide consensus among researchers and the medical establishment that ADHD is a valid diagnosis. However, no definitive neurological cause has been found. There is evidence that supports the etiology of the disorder being related to neurobiological changes [3, 4] and genetic mutations [4, 5], but the scientific community has not ascertained definitively that the disorder arises from a neurological defect, nor have uniform findings been detected among respondents with ADHD [6].

The diagnosis of ADHD is essentially a clinical diagnosis. The physiological and neurological tests for it are incomplete and cannot be considered to be valid “gold standard” tests that can diagnose the disorder unequivocally [7, 8]. The diagnosis of ADHD usually combines clinical observation with subjective reports [9]. The diagnosis and evaluation of children involves obtaining information from different sources, including interviews with parents or teachers, their filling out questionnaires, a clinical evaluation, and if necessary, neuropsychological or other tests [10, 11]. This may lead to overdiagnosis (diagnosis of children who do not actually have the disorder), or under-diagnosis (since the diagnosis often relies on subjective tools, the diagnostician may miss the disorder in a child). This leads to either under- or over-treatment [12, 13]. Therefore, in recent years there has been a substantial effort to develop objective laboratory-based measures to support the clinical diagnosis [14–16].

Since the early 1980s hundreds of studies have explored the biological classification of the disorder. Furthermore, numerous studies tested its treatment with stimulants. Over the years there has been a significant rise in the use of stimulants throughout the world [17, 18], specifically medication such as Ritalin by students in the school system [19, 20]. According to Davidovitch et al. [19], 14.4% of children were diagnosed with ADHD in Israel in 2014, compared to 6.8% in 2005.

Along with studies that indicated the efficacy of administering medication [21, 22], the sharp rise in medication use has sparked concern in international health organizations, because it appears to be administered very easily by professionals even to children who do not definitely need it [23], and due to its short-term risks and side effects [24, 25].

### Parents’ positions and improvement of academic achievements and performance

Johnston et al. [26] found that the best predictor of type of treatment for ADHD is parents’ beliefs about that kind of treatment. Parents who believed in behavior therapy chose it for their children, whereas parents who believed in medication chose it for their children [27, 28]. Research indicates that the education system has a critical impact on reducing parents’ resistance to administering medication [29], and that one of the main reasons for parents’ decision to give their children ADHD medication is improvement of their academic achievements [19, 30]. Although some studies indicate the effectiveness of stimulants and recommend using them as part of a multimodal treatment strategy [31], among whose goals is also improving academic performance [32, 33], there is a scientific dispute over the effectiveness of ADHD medication in improving academic achievement and performance [34–36], even that they have a negative effect [37].

### Marketing ADHD medication to teachers

Many studies indicate that the pharmaceutical companies often use the “third-party technique” to provide their products with scientific cover [38, 39]. The “third-party technique” is a marketing strategy by which pharmaceutical companies deliver their message through experts or organizations that are perceived by the general public as neutral and credible. Most often the manufacturers address doctors in this way, but they approach teachers as well. By providing information, support and guidance to teachers, the companies enlist them to provide positive and supportive information to parents about medication for ADHD.

To market medication, pharmaceutical companies indirectly address teachers through educational websites carrying specific resources for teachers, such as explanations about ADHD and ways to encourage parents to medicate their children for it. Each such website carries a link to the manufacturers website or a link to the stimulant they manufacture. The websites also carry discussions about the diagnosis process and toll-free phone numbers for teachers, school nurses, doctors and lawyers to consult about ADHD and particularly its management at school (for example, http://www.adhdchildhood.com/). Other websites provide scientific-educational online materials for teachers without mentioning specific medications, but they too reinforce the presence of the pharmaceutical companies at the schools. Another strategy is offering education grants to schools for every child diagnosed with ADHD, yielding higher rates of children diagnosed with ADHD and receiving medication [37, 40].

### Teachers’ attitudes and role in ADHD detection and medication

ADHD has been positioned for years as the most common of all chronic neurobehavioral disorders in children [1]. It was found that teachers play a significant role in the initial detection of children with ADHD, on the basis of thier behavior in class [41, 42], and in the diagnosis process and treatment [43–45]. Despite the central role teachers play in referring children for diagnosis, they have very little training about ADHD and its medication [44, 46]. Correspondingly, teachers tend to diagnose children with ADHD at a much higher rate than its frequency according to the DSM-IV [47, 48], frequently support the administration of stimulants to children with ADHD, and point to the effect of medication in improving children’s behavior at school [44, 47]. A study by Havey et al. [48] asked teachers to use the ADHD Rating Scale–IV [49] to rate random students in their classrooms. Results showed that teachers were likely to identify children as having ADHD at rates higher than the expected prevalence rates specified in DSM-IV. Out of 121 rating scales analyzed, 23.97% of students were identified by teachers as meeting criteria for 1 of the 3 types of ADHD [48].

Despite the existing literature about the positive attitudes of teachers and testimonies from the field about the involvement of the education system in the process of identification and referral to diagnosis and medication, we do not know about the characterization of the discourse teachers and counselors conduct with parents about their children’s need to be diagnosed for ADHD and use medication.

### Research objectives

The general objective of the study was to identify and examine the function of the education system in referring school children ages 9–14, suspected to have ADHD, to diagnosis and prescription of medication, based on the reports of teachers, school counselors and parents. The study explored how teachers’ positions influence their discourse with parents about medicating children with ADHD.

The specific research objectives were as follows:

1. To characterize how parents, report the discourse between them and the teachers and counselors about referral of their children to diagnosis and medication.
2. To characterize what messages teachers and counselors use to recommend or not recommend medicating children diagnosed with ADHD with stimulants.

## Method

### Study design

This study used the constructivist qualitative approach, which enables a deep study of the meaning of the experience as it is perceived by the research subjects [50], to examine the experiences of teachers, counselors and parents in the process of referring school children for diagnosis and treatment of ADHD. Constructivist qualitative research is characterized by its holistic approach to the phenomenon [51]. The holistic approach assumes that understanding the context of the phenomenon under study is critical for understanding the reality [52]. The constructivist position views reality as a social construct [53]. In constructivist qualitative research the researcher chooses to use themselves and other people as the main instruments for data collection [54]. The analysis categories arise from the empirical research tools rather than from a priori theories. According to this methodological approach, meaningful hypotheses can only be substantiated after collecting the information. That is, after making contact with the people in the field. In this case, through interviews with parents, counselors and teachers on the issue of ADHD [55].

### Participants

From February 2017 to February 2018 a total of 36 teachers and counselors of school children ages 9–14 (the age group with this highest use of stimulants for ADHD), and 11 parents, from the Jewish and Arab populations were interviewed (Table 1).

**Table 1:** Sociodemographic characteristics of interviewees

Group	Num-ber	Gen-der	Age	Tenure	Proffession	School	Class Grade	Education	Children receiving Ritalin at home
Arab educators	1	F	49	26	Math	Tamra primary	3	BA	None
	2	F	41	16	Sciences	Jdeideh-Makr primary	4–6	BA	None
	3	F	44	22	Math	Tamra primary	5	BA	None
	4	F	45	20	Hebrew	Shefaram primary	4	BA	None
	5	M	40	20	Hebrew and road safety	Jdeideh-Makr primary	6	BA	None
	6	F	48	28	Math	Shaab primary	4	MA	None
	7	F	45	18	Special education	Tamra primary	5–6	BA	None
	8	F	45	20	Special education	Primary Shaab	4–6	MA	None
	9	F	44	20	Arabic	Shaab primary	3	BA	None
	10	F	50	25	Nature and geography	Shefaram primary	5	BA	None
	11	M	43	22	Nature and geography	Shaab primary	5–6	BA	None
	12	F	49	18	Math	Haifa primary	3–5	BA	None
	13	F	59	35	Arabic	Middle school	7	BA	None
Jewish educators	14	F	44	21	Sciences	Nahariya	9	BA	None
	15	F	41	14	History, Jewish culture	Karmiel	7	BA	1
	16	M	40	2	History	Kadouri	7	BA	None
	17	F	55	30	Special education	Karmiel	7	MA	2
	18	F	49	23	Physical education	Kadouri	7	BA	None
	19	M	32	4	History	Misgav	12	BA	None
	20	F	43	18	Math	Misgav	7–12	BA	None
	21	F	46	11	Special education	Karmiel	6	MA	None
	22	M	38	16	Math	Kadouri	7–12	BA	None
	23	F	54	15	Sciences	Migdal Ha’Emek primary	5–6	MA	None
	24	F	32	8	Arithmetic-Engineering	Haifa	3	BA	None
	25	F	37	10	Special education	Kiryat Hayim	Autism class Before 4–6	MA	None
	26	F	33	8	Bible and history	Haifa	Middle school	MA	None
Arab school counselors	27	F	40	14		Shaab primary		MA	None
	28	F	38	15		Jdeideh-Makr primary		MA	None
	29	F	46	7 School counselor 24 Teacher		Jdeideh-Makr primary		MA	None
	30	M	35	10		Shaab primary		MA	None
	31	F	33	8		Shefaram primary		MA	None
	32	F	34	11		Shaab comprehensive		MA	None
	33	F	42	2 School counselor 10 Homeroom teacher		Hula south		MA	None
Jewish school counselors	34	F	46	6 School counselor 13 Homeroom teacher		Misgav middle school		MA	None
	35	F	59	25		Migdal Haemek middle school		MA	None
	36	F	44	5		Middle school Libidor		MA	1
Group	Number	Sex	Age	Years of education	Work	School		Age of child taking Ritalin	Children receiving Ritalin at home
Arab parents	1	F	55	10	–	Shaab		11	1
	2	F	50	12	Cleaning	Shaab		10	1
	3	F	38	BA	Teacher	Shaab		12	1
	4	F	50	12	Assistant kindergarden teacher	Sakhnin		13	1
Jewish parents	5	F	35	12	Cashier	Haifa		11	1
	6	F	41	12	Doesn’t work	Haifa		12	2
	7	F	46	BA	Secretary at high school	Kibbutz Yad Mordechai		12	1
	8	F	46	BA	Emotional therapist	Yokneam area		11	1
	9	F	39	12	–	Nesher		9	1
	10	F	37	12	Self-employed	Haifa		14	1
	11	F	44	BA	Emotional therapist at the Ministry of Education	Tel Aviv		9	1

### Recruitment and procedures

Parents were selected by an intensive heterogenic sampling to represent different ethnicities, ages and socioeconomic statuses [50]. Their initial recruitment was by posting an announcement about the study on forums and social networks (such as WhatsApp). Parents who were interested in participating in the study, left their contact information, and one of the researchers contacted them. In the second stage the snowball technique was used to approach additional parents whose children had been diagnosed for ADHD and who were referred by the participating parents.

In snowball sampling one participant is selected and asked to identify other like individuals who could be added to the sample to understand a phenomenon. Those recruited individuals are asked to identify more people until there are enough participants to understand a phenomenon [56]. The sampling of the school teachers and counselors was deliberately heterogenic. Experienced educators and school counselors were approached based on initial leads. Then a snowball technique was used, with teachers who had been interviewed referring the researchers to their colleagues. The research had some difficulty identifying and recruiting interviewees. Barriers to interviewing parents from the Arab sector surrounded the stigma in Arab society on children with ADHD receiving medication.

Parents we approached from the Arab population asked us to conduct the interviews discreetly because they were concerned about a negative stigma on their child if it became known that they took Ritalin. It is important to note that the Arab community is conservative and has social taboos on subjects such as sexuality, learning disorders and other disorders [57–60]. Following is a quote that demonstrates this concern about a negative stigma, from a mother before her interview. “I want to help you with your study and answer your questions fully and openly, but I want to remind you of the issue of confidentiality because I don’t want anybody from my neighborhood to know that my son takes Ritalin and that you visited me because of something that has to do with him”.

The issue of parents’ fear of stigmatizing their children also came up in interviews with teachers from that community. Following is a quote from one of the teachers: “We kept having to explain and promise to the parents that the question of diagnosis and medication is confidential, and nobody will know anything about it. Sometimes the parent asked us not to tell their children’s other teachers about the diagnosis and medication processes”. Differently, the main barrier to interviews with teachers and counselors from the Jewish sector was lack of time.

To overcome these difficulties, the interviews with school staff were conducted after work hours and outside of school grounds. In addition, we had to offer some of the interviewees (*N* = 18) phone interviews instead of face-to-face interviews.

### Research tools

In-depth interviews were used, based on semi-structured protocols (See Table 2 for example interview protocol with counselors) accommodated to the research population (teachers, counselors, and parents).

**Table 2:** Example interview protocol with counselors

Subject	Questions
Characterization and classification of students	• Please provide me with an overall picture of the kind of children who go to your school. • How would you characterize the different types of children? • What is the common denominator of all of the children with ADHD?
Difficulties and barriers of the teacher	• What difficulties does a teacher have in dealing with a child with ADHD?
Treatment of barriers (opinions)	• How do you think a child with ADHD should be treated? • Do you think teachers have the time and tools to cope? • Have you ever sent a child for an ADHD diagnosis?
Inviting parents for a conversation	• Please characterize different profiles of parents. • When do you invite parents to a talk? Who participates in the conversation with them? Is it you and the referring teacher? • Please describe from the moment when the teacher goes to the school counselor because of the child’s behavior and up to your decision to invite the parents (what are your considerations)? • How do you make the decision to refer a child that you suspect has ADHD to diagnosis? For instance, based on your experience, behavior that indicates a disorder, complaints from subject teachers. • What barriers and concerns do you see in parents whose children need to get diagnosed? • If I were the parent of a child having difficulty in class and I were invited to a talk with you, let’s have such a conversation. What would you tell me? What would you offer me?
Filling out forms	• Who fills out the forms for a child diagnosis by a neurologist? • Do teachers have difficulty filling out such forms? In which parts? Please be specific. • Do you help the teacher fill out the form?
The conversation with the parents after they return with a diagnosis	• How does the conversation between you and the parents go when they are still debating whether or not to take the neurologist’s recommendation to medicate? • Please share with me your emotions during a conversation with parents coming back with a diagnosis. • Let’s say I’m a parent who objects to medication. What would you tell me in the conversation: I will play the parent and you be yourself in the conversation.
Norms	• What is the present norm of the education system in treating children suspected or already having a diagnosis of ADHD? • How frequently do you see children diagnosed with ADHD and receiving medication (Ritalin) among the students in your school?
Counselors’ positions concerning encouraging the use of stimulants	• What do you think of the education system’s approach towards medication for ADHD? • How do you view the rise in the use of medication among students? • What is your position towards other treatment alternatives?
Risk perception	• How do you perceive ADHD, how important is it to treat it and in what ways? • To what extent do you perceive the use of medication as safe and effective? Do you believe it carries risks?
Information sources	• Do you keep yourself informed about ADHD and the different ways to treat it? If so, where do you get that information? • Do you feel a need to receive information from a credible source to keep yourself current about innovations on the disorder and ways to treat it?

### Data analysis

The data was analyzed using content analysis, which attempts to present the full picture by simplification and categorization of the interviews. This analysis was conducted in several stages. The first stage was the initial coding of each subpopulation to create a coding frame so that the themes that arose from the groups of teachers, counselors and parents were integrated separately. The second stage was creating categories based on a number of broad characteristics containing the materials that belonged to that category. The third stage was building the subcategories, considered the smallest information units in the research structure. The fourth stage was an integrated analysis by identifying central themes that arose among the three groups under study [61]. After the data is organized by codes, a description of the data is written, and themes and interrelationships are synthesized. Ultimately tentative answers to research objectives are given, and meanings and themes are described.

The advantage of integrated analysis is that it provides flexibility in the research design by allowing constant movement between the categories and subcategories, to allow the researcher to achieve a design that provides the most comprehensive and credible representation of the studied phenomenon [62, 63].

### Reliability and validity

After conducting pilot interviews, changes were made in the protocols according to the initial findings. All the interviews were recorded and transcribed. The interviews that were conducted in Arabic were transcribed and translated into Hebrew by one of the researchers who is fluent in both languages. In order to validate the research, all of the raw materials were preserved, including recordings of the interviews, transcripts, field diaries and documentation from the collection of raw material, through the various stages of analysis, to the final findings and conclusions [64]. During the data analysis, the researchers read the transcripts and reached consensus regarding the themes. In addition, the selection of the research participants representing different subpopulations (teachers, counselors and parents) reinforces the validity and credibility of the findings.

## Results

First, we will present the educational staff’s main persuasion strategies to persuade parents to have their children diagnosed, followed by a second stage in which we will present the educational staff’s strategies to encourage parents to administer medication (for some selected quotes on the below mentioned themes, please refer to Table 3).

**Table 3:** Selected quotes

The education system’s strategies in convincing parents to have their children diagnosed for ADHD		
The necessity of diagnosis	Parents’ reports of strategy	Teachers and school counselors’ reports of strategy
		“We explain to the parents that we just want a diagnosis, that it is important and necessary to know what the child has and how we can help him, especially because there are a lot of benefits and easements for children with ADHD, and the whole matter of medication does not interest us and we’re not talking about it right now” (teacher from the Arab sector) “Diagnosis is an important step towards identifying the child’s difficulty and does not require medication. Let’s at least identify the problem. You decide for yourself. I’m not telling you to take a pill [...] I’m just asking you to get a diagnosis so at least we’ll know what the trouble is and how to help the child” (a teacher from the Jewish sector)
The child’s best interest	“They said listen, your son needs Ritalin. You have to help him. It is ultimately for his own good. Consult doctors. Doctors usually recommend pills” (a mother from the Jewish sector) “When the teacher told me you have to give your son the medicine because that is how you can help him, it will help him concentrate in class and be a better student, I agreed” (a mother from the Jewish sector)	“You show them and explain to them that it is for the child’s own good, to see where the problem is so we can treat it, to provide a solution to his needs, because there are things we don’t know and he needs to get treated for it, maybe more hours and things like that, we need to understand what the problem is so we can help him” (a teacher from the Jewish sector).
	“When the teacher told me ‘you need to give the boy the medicine because it will help your son, it will help him concentrate in class, be a better student,’ I agreed.” (Mother from the Arab sector).	
Analogies and examples that illustrate ADHD	“She explained that it’s something neurological, a short circuit in the brain, the neurons don’t connect, what that pill does is to connect the neurons and cause a better flow, it allows the brain better absorption.” (a mother from the Jewish sector)	“So I start explaining what it means to have ADHD. And I illustrate, I do a kind of illustration with them. Let’s say you’re holding a weight for a few minutes, then for a few hours and then for a few days, can you hold onto it so long? No, so you drop it. That’s what happens to the kid. He drops his attention. He comes wanting to hold the weight, he comes in the morning wanting to succeed, I believe everybody wants to succeed, but there’s something here that gets in the way and makes it difficult for him.” (school counselor from the Jewish sector) “Unfortunately we have to find techniques and methods to convince parents to get a diagnosis and medicate, even though we know it is not our job and it is a medical matter [...] I explain to the parents that these difficulties are out of the child’s control and it’s not because your son wants to behave that way or he doesn’t want to concentrate, but there is something in your son’s brain, something that holds him back and prevents him from using his abilities.” (school counselor from the Arab sector).
The education system’s strategies in convincing parents to medicate their children		
Improving achievements and academic success	“It is not quiet I’m looking for. I’m interested in my child’s achievements in school. Focus, focusing on the question, willingness to learn, the availability to learn improves fivefold. I tell parents this is my experience. I say to the mother, send him to a test without Ritalin and see the result, instead of 100 he might get 50. It’s something that makes the child focused.” (teacher from the Jewish sector) “The teacher and counselor told me that if I gave my son medication he could get higher grades on the tests, which would help prevent him from flunking, especially because he had bad grades.” (a mother from the Arab sector)	
Improving the child’s functioning socially and behaviorally, improving their self-image and providing an experience of success.	Socially: “Children with ADHD are usually perceived in the class as nuisances. They disrupt the class and label themselves in such a way that the class perceives them as disrupting learning. They interrupt a lot and have hyperactive behaviors that antagonize the other children who are not tolerant of this and do not know how to tolerate hyperactivity, chatter, impulsiveness, and therefore they shun that kid” (school counselor from the Jewish sector)	
	Behaviorally: “Very soon he will go to middle school and high school. Here he is still among little kids. In high school there are kids who are bigger than him. He will be exposed to smoking and drugs and you will not be able to control him. Let him become ready to move from here to high school. Give him the tools he needs to take care of himself and not go near such things. Hyperactivity influences behavior and everything in life. If you treat it with medicine the child will calm down and be restrained in his behavior too. The medicine definitely does not only influence school achievements.” (A homeroom teacher from the Arab sector)	
	Improving self image. “Ritalin helps the child concentrate better, his grades go up, the child starts to feel better. When he sees that he understands what the teacher is explaining and the teacher praises him, after all of the frustration he went through before the treatment, his classmates except him more, his grades go up, it all improves his self-image and he starts working harder and even taking initiative.” (guidance counselor from the Arab sector)	
	Experience of success for the child**:** “I say that relative to what he has experienced until now, failed grades, failed behavior, I say this is what we are trying to produce. We want to give the child an experience of success now, not in 10 years, because the moment he experiences success, belief in himself is the first thing.” (a teacher from the Jewish sector)	

All told, it was found that the word “persuasion” appeared 68 times in the interviewees’ texts of both stages. Only a small number of the counselors and teachers (12%) reported that “it is not their job to influence parents’ decision to have their children diagnosed for ADHD and medicated.”

### The education system’s strategies in convincing parents to have their children diagnosed for ADHD

#### The necessity of diagnosis

89% of the Jewish and Arab educators and counselors suggest diagnosis as a necessary first step to help the child, to determine the most suitable treatment approach and to be able to utilize the various support resources the school offers. Counselors often mentioned the importance of distinguishing between diagnosis and medication, particularly when they were met with parents’ resistance.

#### The child’s best interest

89% of teachers and counselors interviewed used the concept of “the child’s best interest” in order to explain to the parents the importance of diagnosis. According to them, diagnosis would help children who do not fit in in the classroom, who are unable to utilize their talents, and pay a heavy price both academically and socially. It is noteworthy that this theme also came up in the interviews with parents (64%), and they reported that that argument had an influence on their decision to medicate.

#### Analogies that illustrate ADHD

In 15% of the interviews with educators and counselors they mentioned that they had decided to explain to the parents in “popular” language what ADHD is, and used analogies from everyday life to illustrate it, such as: what happens to a child carrying weights without support, or to a child who needs glasses but does not have them. Some of the teachers contradicted themselves by saying that they did not get into medical discussions with parents, and then illustrated with analogies how they explained the disorder to parents.

### The education system’s strategies in convincing parents to medicate their children

#### Medication improves the child’s performance

85% of the teachers and counselors said they explained to the parents that medication improves the child’s performance academically, socially, emotionally and/or behaviorally. Some of the educators and counselors (36%) said they explained to parents that medication can help their child concentrate and focus in school and be available to learn and develop their talents in order to improve their grades. Many of the educators and counselors (63% in the Arab sector, and 38% in the Jewish sector), believed that that strategy had a very big influence on parents’ decision to medicate their children. Furthermore, 42% of educators and counselors reported that giving an anonymous example of children who received medication and as a result improved their school performance had a big influence on parents’ decision to medicate their children. Accordingly, in the interviews with parents, 64% mentioned that that strategy did indeed encourage them to medicate their children, because they claimed every parent wants their child to succeed and excel at school. As for improving the child’s social and behavioral functioning with medication, 45% of the educators and counselors tried to convince parents to medicate their children by presenting the negative consequences of not medicating them: they said a child who is untreated disrupts the class and bothers other children, and their classmates and the school staff prefer to stay away from them, compared to children who take medication and experience social and behavioral improvement and a boost in their self-image. 54% of them also told parents that medication gives children a high feeling of success and self-efficacy. Finally, use of fearmongering was found as an additional strategy. Teachers and counselors (31%) said that they warned against the negative consequences of failing to take medication such as transfer to a special education class (11%), dropping out of school (14%), failing to be promoted to the next grade (8%), and suspension from school (6%), which would undermine the child’s coping within the education system. Accordingly, 36% of the parents who were interviewed admitted that their fear of a special education class was one of the considerations they took into account in their decision to medicate their child.

#### Normalization of medication

45% of the educators and counselors reported that they tell parents that taking medication is common and normative, and thereby reduce parents’ fears and concerns about the stigma. Parents (64%) reported that educators told them that not only their child needed medication but many children in many schools receive medication for ADHD.

#### A role model who takes medication

54% of the teachers and counselors demonstrate the positive contribution of medication through examples of celebrities and successful members of society, who also use such drugs. Parents (36%) reported that educators and counselors had explained to them that people with higher education, who are successful in life and serve in senior positions, also take medication.

#### ADHD as a chronic disease

20% of the teachers and counselors referred to ADHD as a disease like any other chronic disease (such as diabetes or hypertension).

#### The medication causes no harm

22% of the educators and counselors reported that they overcome parents’ fear of damage to their children’s health by explaining that the drug does not cause dependency or addiction and has no harmful effects currently known to science. 39% of the teachers and counselors reported that they refer parents to scientific studies or doctors to convince them of the drug’s safety. In addition, 75% of the teachers and counselors from the Jewish sector refer parents to academic studies and articles to convince them of the safety of the medication, compared to 10% of the teachers and counselors from the Arab sector.

#### Reporting the child’s negative behaviors

In 51% of the interviews with the educators and counselors it emerged that reports of children’s negative behaviors in school influenced parents. Accordingly, most of the parents who were interviewed (73%) mentioned the high frequency of calls from their children’s teachers and the many recurring complaints as a factor that affected their decision to give their child medication.

#### Sharing examples from the teachers’ and counselors’ personal lives with parents

Teachers and counselors who had a child with ADHD in their family who was treated with medication, shared their personal experiences with the parents. They noted that sharing their personal experiences promoted the parents’ decision to give their child medication.

#### The child’s will

12% of the teachers and counselors reported that they asked the child to describe the academic and behavioral difficulties they experienced.

## Discussion

According to the guidelines of the Israeli education system, medication for children with ADHD is under the sole responsibility of neurologists, psychiatrists and pediatricians, and educational staff do not have the authority to offer it or interfere with it. The study findings indicate that, nonetheless, educational staff initiate and become influential factors in parents’ decision-making.

It was found that educators and counselors use persuasion strategies in attempting to influence parents’ beliefs, attitudes, intentions, motivations, or behaviors, specifically using systematic persuasion strategies to encourage parents to have their children diagnosed and treated for ADHD. The interviewees used the word “persuasion” 68 times when describing the different ways to promote the diagnosis and administration of medication. These findings correspond with the literature, according to which teachers have positive attitudes towards medicating ADHD children with stimulants, and indicate their efficacy in improving children’s behavior at school [44, 47].

Key findings from the present study indicate two main interventions by the educational staff in ADHD: one is encouraging diagnosis, and the other is encouraging medication. In both of these interventions the teachers and counselors, according to the parents’ reports and according to their own reports, use a variety of persuasion strategies that affect parents’ decision-making. The interviews indicate that parents are in no hurry to medicate their children, and in most cases mount initial resistance to the idea. This finding corresponds with the literature, according to which parents prefer non-medication therapeutic options [29], and tend not to comply with medication with stimulants [65, 66]. Therefore, teachers and counselors in the present study often mentioned the importance of distinguishing between diagnosis and medication. They made this distinction especially when parents objected to medicating their children.

It can be argued that the educational staff employed persuasion strategies known as “foot in the door,” a way to achieve behavioral compliance with minimum pressure [67]. Accordingly, teachers and counselors in the present study acted to convince the parents first only to get a diagnosis, which draws less resistance, while emphasizing the difference between it and medication. Only once the child was diagnosed with ADHD did the educational staff (85% of the teachers and counselors interviewed) transition into a discussion of medication, persuading parents by framing the medication not only as a medical solution but also as a wellbeing solution, which would provide the child with emotional and physical wellbeing as part of a multimodal treatment strategy [31, 68] to improve their achievement and academic success. This strategy resembles the pharmaceutical companies’ branding strategy used to frame other drugs [69]. According to Hansen [70], one of the main causes of parents’ surmounting their objection to medication is their anticipation of its positive influence their children’s academic performance and relationships with peers and staff. However, it should be noted that evidence of the impact of medication on the improvement of academic achievements of children with ADHD is in scientific dispute [35, 36].

In addition, the educators and counselors use the strategy of normalization to encourage parents to medicate their children, arguing that medications for ADHD have become common in society. One of the most effective persuasion techniques is referral to social norms [71–74]. Social norms are typically defined as “rules and standards that are understood by members of a group, and that guide or constrain social behaviors without the force of law” [75], and often relate to a perceived social pressure to engage or not engage in specific behaviors [76]. Perceived normative peer behaviors and attitudes have emerged as key predictors of health behaviors, and it is norms that are largely responsible for health behaviors. According to Nolan et al. [77], normative beliefs are more predictive of behavior and behavior change than any other beliefs. In the present study, teachers and counselors said that they explained to parents that medication for ADHD is a normative and accepted solution among other children and even among famous people.

As soon as medication is normalized, there is a risk that children who do not need it will be medicated [78, 79]. We do not wish to argue that the drug should become illegitimate but simply that it should only be given to children who medically need it.

The teachers and educators in this study made an effort to mitigate parents’ concerns about medication, and to that end also illustrated its positive contribution using examples of celebrities and successful people as role models who themselves use such medication. It is known that exposing celebrities in the media effective in influencing the public’s positions, beliefs and behaviors concerning health [80], as they serve as positive role models for society and guide the aspirations and goals of many others [81, 82]. According to parents’ reports, teachers and counselors are also perceived as positive role models, and that is why sharing with parents the educators’ personal experiences of raising a child with ADHD increased parents’ reported compliance with medication for their children.

One of the dominant strategies that arose from the study is the negative framing of the consequences of failing to take stimulants for ADHD. Some of the interviewed parents mentioned the high frequency of calls they received from teachers and the many complaints about their children as factors that influenced their decision to give the medication. In addition, the educators and counselors chose to tell the parents that the drug does not cause dependency or addiction and has no damaging effects currently known to science. This information is inconsistent with the fact that the medication in question is a narcotic which is considered a drug, and with numerous studies that have found that stimulants carry short-term risks and side effects [24, 25, 83, 84].

### Limitations

The research limitations are that since this was a qualitative study it did not use a representative sample of the teachers and counselors operating in the Israeli education system or the parents whose children take stimulants. However, the in-depth interviews expose the involvement of the education system in the decision-making process, raising numerous ethical and professional issues.

## Conclusions

This study does not distinguish between children diagnosed with ADD alone and those who are also diagnosed as hyperactive. Follow-up studies might examine whether the strategies used by the counselors and educators are different between those two groups.

The present study found that the educational staff in the schools are involved in parents’ decision-making process to medicate their children and use various persuasion strategies to encourage parents to diagnose and medicate to treat ADHD, which contradicts the education system’s guidelines. School is supposed to serve as a gatekeeper for parents, and therefore it is important for the ministry of education to set clear professional and ethical limits. Likewise, it is the responsibility of the schools to deal with different kinds of children’s behaviors and offer different alternatives and accommodated supportive educational settings.

## Abbreviations

ADHD: Attention Deficit Hyperactivity Disorder
DSM-IV: Diagnostic and Statistical Manual of Mental Disorders, 4th Edition
